# Antioxidative Potentials of Incretin-Based Medications: A Review of Molecular Mechanisms

**DOI:** 10.1155/2021/9959320

**Published:** 2021-04-27

**Authors:** Habib Yaribeygi, Mina Maleki, Thozhukat Sathyapalan, Tannaz Jamialahmadi, Amirhossein Sahebkar

**Affiliations:** ^1^Research Center of Physiology, Semnan University of Medical Sciences, Semnan, Iran; ^2^Chronic Kidney Disease Research Center, Shahid Beheshti University of Medical Sciences, Tehran, Iran; ^3^Academic Diabetes, Endocrinology and Metabolism, Hull York Medical School, University of Hull, UK; ^4^Department of Food Science and Technology, Quchan Branch, Islamic Azad University, Quchan, Iran; ^5^Department of Nutrition, Faculty of Medicine, Mashhad University of Medical Sciences, Mashhad, Iran; ^6^Biotechnology Research Center, Pharmaceutical Technology Institute, Mashhad University of Medical Sciences, Mashhad, Iran; ^7^Applied Biomedical Research Center, Mashhad University of Medical Sciences, Mashhad, Iran; ^8^School of Pharmacy, Mashhad University of Medical Sciences, Mashhad, Iran

## Abstract

Glucagon-like peptide 1 receptor agonists and dipeptidyl-peptidase 4 inhibitors are medications used for managing diabetes, mimicking the metabolic effects of incretin hormones. Recent evidence suggests that these medications have antioxidative potentials in the diabetic milieu. The pathophysiology of most diabetic complications involves oxidative stress. Therefore, if incretin-based antidiabetic medications can alleviate the free radicals involved in oxidative stress, they can potentially provide further therapeutic effects against diabetic complications. However, the molecular mechanisms by which these medications protect against oxidative stress are not fully understood. In the current review, we discuss the potential molecular mechanisms behind these pharmacologic agents' antioxidative properties.

## 1. Introduction

The incidence of diabetes mellitus (DM) is increasing in epidemic proportions globally [1]. DM carries considerable morbidity and takes up a significant proportion of health care burden and costs [2, 3]. DM gives rise to various microvascular and macrovascular complications [4]. The development of diabetic complications involves different pathophysiologic pathways such as polyol pathway, hexosamine pathway, inflammatory responses, oxidative pathways, peroxidation, glucotoxicity, and lipotoxicity [4, 5]. Although the exact pathophysiology of various diabetic complications is not clearly understood, there is growing evidence that oxidative stress plays a crucial part [6]. Hence, many antioxidative agents have been tried for treating DM and its complications [7]. Glucagon-like peptide receptor-1 agonists (GLP-1RA) and dipeptidyl peptidase-4 inhibitors (DPP-4i) are classes of antidiabetic medications that are used in the management of DM mimicking the action of incretin hormones [8, 9]. They reduce hyperglycemia through several mechanisms [8, 10].

In addition to their antihyperglycemic effects, recent evidence suggests that these medications could provide antioxidative effects [11, 12]. If they can ameliorate DM-induced oxidative stress involved in the pathophysiology of diabetic complications, they could offer potential additional therapeutic effects in the management of diabetic complications [12–14]. However, the exact molecular mechanism by which these antidiabetic medications reduce oxidative stress is not fully elucidated. This review discusses the antioxidative properties of the incretin-based antihyperglycemic agents and the potential molecular mechanisms behind these effects. We conducted an extensive literature search using keywords of oxidative stress, diabetes mellitus, glucagon-like peptides, GLP-1 receptor agonist, and DPP-4 Inhibitor in databases such as PubMed, Scopus, the Web of Science, and Google Scholar between 2002 and 2020. However, the main limitation was the lack of enough related evidence. Still, we selected appropriate literature by extensively reading the available evidence and finding the most appropriate one demonstrating the possible involved molecular pathways.

## 2. Classifications of Diabetes Mellitus

DM is typically categorized into type 1 and type 2 diabetes [15]. Type 1 DM (T1DM) is mainly referred to as lower circulatory insulin due to beta cell failure [15]. Type 2 DM (T2DM), which is the most prevalent form of DM, is mostly related to insulin resistance in insulin-dependent peripheral tissues [15]. Gestational diabetes is another form of DM which occurs in pregnant women likely due to hormonal variations during pregnancy [16]. In addition, there are other types of DM such as LADA (latent autoimmune diabetes in adults), maturity-onset diabetes of the young (MODY), secondary diabetes due to various pathological conditions such as pancreatitis, and secondary diabetes to certain medications, e.g., corticosteroids [17, 18]. As described below, GLP-1 mimetics exert antidiabetic effects in both type 1 and type 2 DM [10].

## 3. GLP-1RA and DPP-4i

GLP-1RA is a class of antidiabetic medications that provide antihyperglycemic effects by mimicking incretin hormones' effects through stimulation of GLP-1 receptors [19, 20]. Incretin hormones are a group of intestinal metabolic peptides such as GLP-1 and GIP (gastric inhibitory peptide), which reduce hyperglycemia via several pathways, including inhibition of glucagon, stimulating insulin secretion, delaying the gastric emptying, appetite suppression, declining intestinal nutrients absorption, improvement of lipid metabolism, and promoting pancreatic *β*-cell efficiency [19, 21–24]. These medications have a specific receptor known as the GLP-1 receptor, a member of G-protein coupled receptors, which are principally located in surfaces of pancreatic *β*-cells [22, 25]. GLP-1R activation is generally followed by cAMP (cyclic adenosine monophosphate) production leading to cellular depolarization and insulin secretion from the pancreatic *β*-cells [22, 26]. GLP-1 is commonly metabolized by a protease known as dipeptidyl peptidase-4 (DPP-4), and thereby, DPP-4 inhibitors (i) provide antihyperglycemic effects by increasing the active levels of GLP-1 [27, 28]. Hence, the DPP-4i has similar but less potent effects than GLP-1RA (Table 1) [27].

### 3.1. Oxidative Stress and Diabetic Complications

Free radicals are active molecules that have unpaired electron(s) in the outer layer of their orbitals, which enables them to bind with an unpaired electron(s) in other molecules as well as biological particles [29]. These active species are generated physiologically through various biologic events and have physiologic roles in specific cellular processes [29]. The components of the antioxidative system neutralize the excess amounts of free radicals [29]. When the production of free radicals is increased or when the antioxidative system is weakened due to conditions such as DM, a pathologic state of oxidative stress is developed. Oxidative stress develops when free radicals overcome the ability antioxidative system to neutralize excess free radicals [29, 30]. These excess free radicals will interfere with various physiological states, including complications resulting from diabetes [30]. Hence, the prevention of oxidative stress and improving redox state towards physiologic balance is crucial for preventing the development and progression of various disease states, including DM and its complications [31–34].

In addition to the direct deleterious effects on biologic elements, oxidative stress is a potent upstream event for various molecular pathways such as apoptosis, necrosis, autophagy, synthesis/release of nitric oxide, renin-angiotensin system (RAS), inflammatory responses, and autonomic nervous system [30, 35]. It has bidirectional interactions with these molecular pathways, including those pathways involved in the development of diabetic complications [6, 36]. Oxidative stress initiates a cascade of events, resulting in the dysfunction of various tissues [6]. For example, excess amount of free radicals upregulate many types of inflammatory mediators such as tumor necrosis factor-alpha (TNF-*α*), transforming growth factor-beta (TGF-*β*), interleukins (ILs), monocyte chemoattractant protein-1 (MCP-1), matrix metalloproteinase (MMP), nuclear factor-kappa b (nf-*κ*b), E-selectin, chemokines, and different forms of adhesion molecules [37–39]. These procytokines are involved in the pathophysiology of the development of diabetic nephropathy [6, 40]. Also, oxidative stress stimulates the expression and activity of apoptotic mechanisms such as p53, Bax/Bcl2 ratio, and caspases, leading to a higher rate of cellular apoptotic death [41, 42]. Moreover, oxidative stress has potent direct effects on various biologic molecules such as peptides, lipids, carbohydrates, and, more importantly, nucleic acids, thereby negatively modifying their physiologic structures and function [43, 44]. Hence, oxidative stress has significant roles in developing and progressing various diabetes-induced complications, including nephropathy, retinopathy, neuropathies, and cardiovascular diseases [31, 36, 45, 46].

## 4. Antioxidative Potentials of GLP-1RA and DPP-4i

**Figure 1:**
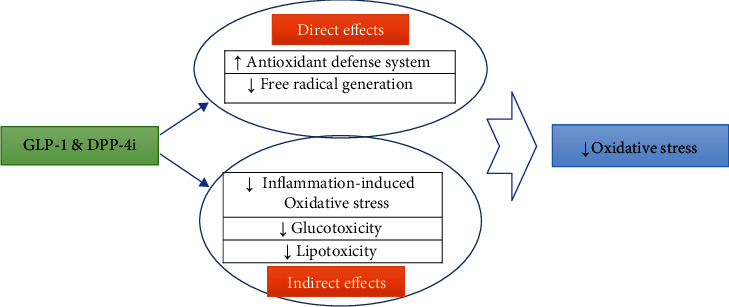
Possible antioxidant effects of GLP-1 receptor stimulation.

There is growing evidence suggesting incretin-based antidiabetic medications can normalize the redox state in an oxidative milieu [47–49]; however, all molecular mechanisms involved are not elucidated [48]. In the following sections, we will review these interconnected molecular pathways (Figure 1; Table 2).

## 5. Direct Effects

### 5.1. Antioxidant Defense System: Roles for Nrf2, Sirt-1, and Sirt-3

Antioxidant defense system (ADS) is an intrinsic molecular structure in most types of eukaryotic cells that neutralizes the different forms of free radical species, attenuates their harmful impacts, and protects against oxidative damages [50]. This protective system consists of both enzymatic and nonenzymatic elements such as superoxide dismutase (SOD), catalase (CAT), glutathione reductase (GR), and glutathione peroxidase (GPX), which are effective free radical scavengers and, thereby, their concentration, as well as activity, is a significant determinant of redox state in biologic milieu [51, 52]. Therefore, any agent that will be able to potentiate ADS will ameliorate various oxidative stresses and reduce the oxidative damages [7, 31, 50].

There is growing evidence to suggest GLP-1 receptor induction potentiates the ADS through several pathways [53–55] [53–56]. Wang et al. in 2017 reported that after 24 hrs of GLP-1 therapy in human umbilical vein endothelial cells (HUVEC), cultured cells reversed the increase of high glucose-induced oxidative markers such as malondialdehyde (MDA) and oxidized LDL (ox LDL) via upregulation of NQO1 (NAD(P)H Quinone Dehydrogenase 1), HO1 (Heme oxygenase 1), and GPX genes [53]. Induction of diabetes milieu in HUVECs is currently used in many studies [57]. In this design, HUVECs are cultured in a high glucose medium (i.e., 33 mM glucose for about 72 h at 37°C) to induce DM milieu [57]. Civantos et al. in 2017 found that sitagliptin attenuated DM-induced oxidative stress in renal tissues by the potentiation of ADS via miR-200a dependent pathway in diabetic rats [55]. Moreover, Shiraki and coworkers in 2012 observed that liraglutide increases the SOD-2, catalase, and GPX expression levels and thereby reduces TNF-*α* induced oxidative stress in the cultured HUVEC cell line [58]. Mangmool and coworkers in 2015 showed that exendin-4 upregulates ADS elements of CAT, GPX, and MnSOD in a dependent manner to Epac (exchange protein activated by cAMP) in cardiomyocytes of rats [59]. Bułdak and colleagues in 2015 reported that exenatide potentiated the antioxidative capacity of cultured human leukocytes increased expression of ADS elements [60]. Mohiuddin and coworkers in 2019 provided similar evidence in neuronal networks confirming GLP-1 receptor induction ameliorates oxidative damage in immortalized cultured dorsal root ganglions in the diabetic milieu [61]. This evidence demonstrates that GLP-1 receptor induction can potentiate ADS [54, 59–62].

Nuclear factor erythroid 2-related factor 2 (Nrf2) is a member of the basic leucine zipper (bZIP) nuclear transcription factors family, which enter the nucleus after phosphorylation, form heterodimers with other regulatory proteins, and bind to the specific regulatory regions of DNA known as ARE [63]. This factor is responsible for controlling antioxidant proteins' expression, thereby playing an essential role in keeping a normal redox state in cells [63]. Genetic knockdown of these transcription factors makes the tissues more vulnerable to oxidative injuries [64]. Also, pharmacologic activation of Nrf2 activity potentiates the ADS and protects tissues against oxidative damages [65–67].

We have evidence suggesting GLP-1 receptor induction stimulates the Nrf2 signaling pathways in various tissues [11, 68]. For example, Deng and coworkers in 2018 found that liraglutide induces Nrf2 signaling pathways and increased the expression level and activity of Nrf2 protein in the brain of diabetic rats [68]. Also, Fernández-Millán et al. in 2016 observed that GLP-1 increased the Nrf2 expression and activity in pancreatic islets [69]. Moreover, Kim and colleagues in 2017 established that exendin-4 activates the Nrf2 signaling pathway and potentiates ADS element in rat insulinoma cells [70]. This evidence strongly suggests that GLP-1 receptor induction can stimulate Nrf2 signaling leading to an improved redox state [68–70].

Sirtuin (sirt) is a family of highly conserved proteins in mammals with at least seven members as Sirt1-7 [71]. Although these proteins' exact functions are not understood, some members of this family are involved in cell survival, life span, and response to various stimuli such as oxidative stress [71, 72]. Recent evidence demonstrated that Sirt-1 and Sirt-3 play a significant role in diabetes-related disorders and oxidative stress [71, 73]. Sirt1 is a NAD- (nicotine-amide adenine-)^+^ dependent deacetylase that controls oxidative stress responses and apoptosis via a p53 dependent mechanism, and recent studies suggest that its activity is decreased in DM [74, 75]. Sirt3 is another member of the sirtuin family, which is also involved in the oxidative response, especially in mitochondria, and controls metabolic pathways via NAD^+^-dependent deacetylase [76, 77].

There is evidence to suggest that incretin-based antidiabetic medications have interactions with sirt proteins [73, 78, 79]. GLP-1 receptor activation may increase or decline Sirt-dependent pathways in various conditions [80]. Zheng and coworkers in 2017 found that exenatide improved endoplasmic reticulum (ER) stress via sirt1 dependent pathway in hepatic cells of C57BL/6J mice [81]. Lee et al. in 2012 reported that exendin-4 upregulates sirt1 in hepatic cells of high-fat diet-dependent obese mice [82]. These findings suggest that GLP-1RA and DPP-4i can provide some antioxidative effects via sirt proteins. For example, Zeng and coworkers in 2016 demonstrated that exendin-4 improved oxidative stress by promoting ADS potency via Sirt1 and sirt3 upregulation in the retina of diabetic rats [73]. The other possible molecular pathways, such as cAMP/PKA/ERK, have also been suggested by which GLP-1 upregulates the ADS elements [69]. It has been suggested that the Nrf2 signaling pathway can be also activated via the cAMP/PKA/ERK pathway [69]. Fernández-Millán and coworkers in 2016 found that GLP-1 increases antioxidant capacity and prevents oxidative damages via inducing Nrf2 protein expression and its translocation in a cAMP/PKA/ERK-dependent manner in diabetic beta cells [69]. So, GLP-1 mimetics are able to potentiate the activity of ADS elements via several molecular mechanisms.

### 5.2. Free Radical Generation

**Figure 2:**
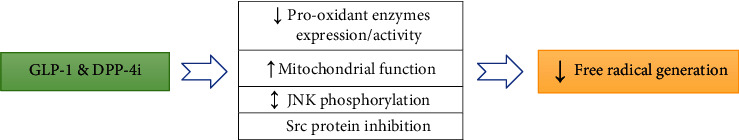
GLP-1 receptor induction reduces free radical species through four main molecular pathways (JNK: Janus kinase; Src: an adaptor protein).

GLP-1 mimetic can reduce free radical generation through several pathways (Figure 2):

#### 5.2.1. Prooxidant Enzymes

GLP-1 receptor activation has inhibitory effects on prooxidant enzymes and reduces their expression and activity [54]. Shiraki and colleagues in 2012 demonstrated that liraglutide downregulated the gp91^phox^ and p22^phox^ subunits of NADPH (nicotinamide adenine dinucleotide phosphate oxidase) oxidase (Nox) in cultured HUVECs [54]. This effect was accompanied by lower free radical generation and improvement in oxidative stress [54]. Similarly, Bułdak and coworkers in 2015 found that exenatide reduces free radical species via downregulation of NADPH oxidase in cultured human leukocytes [60]. Moreover, Li and colleagues in 2017 reported that pretreatment of HUVEC cells with GLP-1 reduces the p47^phox^ subunit of NADPH oxidase-4 and reduces DM-induced free radical generation [83]. Choi and colleagues in 2017 provided further evidence indicating gemigliptin (DPP-4i) and reduced mRNA expression of p22^phox^ subunit of Nox, leading to lower free radical generation in rat model of adenine-induced chronic kidney disease [84].

#### 5.2.2. Mitochondrial Dysfunction

Mitochondrial dysfunction is commonly associated with higher amounts of free radical production and oxidative stress [85]. There is some evidence to imply that GLP-1 receptor induction may improve mitochondrial function [80, 86]. Kang et al. in 2015 demonstrated that GLP-1 stimulates mitochondrial biogenesis, increases the mitochondrial membrane potential, and improves mitochondrial function in INS-1 (rat insulinoma cells) [86]. Góralska and coworkers in 2017 found that exendin-4 markedly improved mitochondrial efficiency in human adipocytes [80]. Also, Zhang and coworkers in 2017 demonstrated that alogliptin (DPP-4i) promoted mitochondrial function and attenuated mitochondrial free radical production in atrial tissues of diabetic rabbits [87].

#### 5.2.3. Other Pathways

GLP-1RA and DPP-4i may reduce free radical generation through other molecular pathways [88, 89]. Laviola et al. in 2012 has reported that GLP-1 decreased free radical species by a JNK- (c-Jun N-terminal protein kinase-) dependent pathway and avoiding JNK phosphorylation in cultured human cardiac progenitor cells [89]. Mukai and coworkers in 2011 provided evidence indicating exendin-4 decreases endogenous free radical species by inhibition of Src protein in islets of diabetic rats [88].

## 6. Indirect Effects

### 6.1. Inflammation-Induced Oxidative Stress

There is growing evidence that inflammatory responses can induce oxidative stress [49, 90]. Inflammation-dependent oxidative damage is a known event in the inflamed tissues [90–92]. GLP-1R induction may ameliorate these phenomena [54, 93]. Shiraki and colleagues in 2012 stated that liraglutide suppresses TNF-*α* induced oxidative stress in cultured HUVEC cells [54]. They found that treatment with liraglutide markedly reduced inflammatory cytokines, which, in turn, attenuates inflammation-induced oxidative markers as MDA and oxLDL [54]. Alam et al. in 2015 reported that sitagliptin inhibited inflammation-induced oxidative damages in renal tissues of rats [49].

### 6.2. Glucotoxicity

Glucotoxicity refers to the toxic effects of hyperglycemia [94, 95]. It can induce and exacerbate oxidative stress in several ways, including increasing the free radical species such as AGEs (advanced glycation end-products) and weakening the potency of ADS potency. An improvement in glucose homeostasis translates to a balanced redox state [94, 96]. GLP-1 mimetics can improve glucose homeostasis and attenuate glucotoxicity by amplifying insulin signal transduction [97–99]. We have shown before that these agents can improve the insulin signaling pathway [10] through several mechanisms such as regulation of glucose transporters in 3T3-L1 adipocytes [100], upregulation of phosphorylated IR-*β*, IRS-1, Akt, and GSK-3beta (Glycogen synthase kinase 3 beta) in adipocytes [101], promoting Akt phosphorylation and cyclins A, D1, and E protein expression in adipocytes [102], and reducing toxic byproducts of AGEs leading to better glucose homeostasis in diabetic milieu [103, 104]. Beyond the pathways mentioned above, they can indirectly normalize the redox state by promoting glucose metabolism [97, 98].

### 6.3. Lipotoxicity

Lipotoxicity occurs in patients with diabetes, mainly referring to altered lipid metabolism leading to a higher production of toxic byproducts such as MDA, which is the primary marker of oxidative damage [105]. GLP-1 receptor activation can improve lipid metabolism and reduction of toxic metabolites [106–108]. They can exert these metabolic effects in several ways, such as peroxisome proliferator-activated receptor alpha (PPAR-*α*) [109]. This evidence suggests that incretin-based antidiabetic medications can reduce oxidative stress by improving lipid metabolism [108, 109].

## 7. Clinical Evidence

There is some clinical evidence to show that these medications protect against oxidative damages [12, 110] (Table 3). Bunck and coworkers in 2010 demonstrated that GLP-1 reduces oxidative markers such as MDA and oxLDL in patients with T2DM [110]. Also, Ceriello and coworkers in 2013 reported that GLP-1, through its antioxidative potentials, protects against endothelial dysfunction in patients with T1DM [12]. Moreover, Okada and colleagues in 2014 conducted a clinical trial on patients with T2DM, demonstrating that liraglutide provides cardioprotective effects via its antioxidative potentials [111]. Furthermore, Rizzo and coworkers in 2015 found that liraglutide attenuated oxidative stress markers in patients with T2DM [112] (Table 3).

## 8. Conclusion

GLP-1 mimetics improve oxidative stress through various direct and indirect pathways (Figure 1, Table 2). Direct antioxidative effects of incretin-based drugs are through their impact on ADS and free radical generation. They can potentiate the ADS by increasing the expression and activity of its components. They can also reduce free radical species through several pathways, such as prooxidant enzymes and mitochondrial function. These medications can also attenuate oxidative stress via indirect mechanisms such as lowering glucotoxicity, lipotoxicity, and inflammation-dependent oxidative stress. However, the limitations include a lack of enough direct experimental and clinical evidence confirming our suggested pathways, and, thereby, there is a need for more studies to confirm these effects. Especially, more clinical evidence is required to validate the findings of related experimental studies. Besides, potentially more molecular pathways involved in these effects may be discovered in the near future.

## Figures and Tables

**Table 1:** Two main classes of incretin-based antidiabetic medications.

	Approved drugs	Mechanisms of action	Ref.
GLP-1ra	Exenatide (exendin-4), albiglutide, liraglutide, lixisenatide, semaglutide, dulaglutide	Mimic hypoglycemic influences of incretin hormones	[19, 20]
DPP-4i	Sitagliptin, saxagliptin, vildagliptin, linagliptin	Increase the active circulatory levels of GLP-1	[27, 28]

**Table 2:** Possible molecular pathways by which incretin-based antidiabetic medications protect against oxidative damages (SOD: superoxide dismutase; CAT: catalase; GPX: glutathione peroxidase; Nrf2: nuclear factor erythroid 2-related factor 2; AGEs: advanced glycation end products; Sirt: sirtuin; MDA: malondialdehyde).

	Molecular mechanism	Effects on oxidative stress	Ref.
Direct roles	Antioxidant defense system	Increase expression/activity of antioxidative elements such as SOD, CAT, and GPX at least partly via Nrf2 and Sirt signaling pathways	[60, 68, 69, 73]
	Free radical generation	Reduce the free radical generation thru several pathways such as suppressing prooxidant enzymes and improving mitochondrial function	[54, 60, 86]
Indirect roles	Inflammation-induced oxidative stress	Attenuate procytokines' expression/release leading to inflammation-induced oxidative stress	[49, 54]
	Glucotoxicity	Improve insulin signaling as well as glucose homeostasis leading to lower amount of toxic byproduct as AGEs	[97, 98]
	Lipotoxicity	Reduce lipid metabolites such as MDA due to promoting lipid metabolism	[106–108]

**Table 3:** Clinical evidence about the antioxidative potentials of GLP-1RA and DPP-4i.

Treatment	Population of study	Effects	Ref.
Exenatide	69 patients with T2DM	Reduced oxidative markers as MDA and oxLDL	[110]
GLP-1	15 patients with T1DM	Restored oxidative stress in endothelial cells, improved endothelial function	[12]
Liraglutide	64 patients with T2DM	Provided cardioprotective effects via attenuating oxidative stress	[111]
GLP-1	60 patients with T2DM	Improved the palmitate-induced oxidative damages in cardiomyocytes	[113]
Liraglutide	20 patients with T2DM	Declined oxidative markers and free radical generation	[112]

## Data Availability

Not applicable.
